# Emerging Roles of MicroRNAs in EGFR-Targeted Therapies for Lung Cancer

**DOI:** 10.1155/2015/672759

**Published:** 2015-07-26

**Authors:** Fei Han, Jinxi He, Feng Li, Jiali Yang, Jun Wei, William C. Cho, Xiaoming Liu

**Affiliations:** ^1^Human Stem Cell Institute of the General Hospital, Ningxia Medical University, Yinchuan, Ningxia 750004, China; ^2^Department of Thoracic Surgery of the General Hospital, Ningxia Medical University, Yinchuan, Ningxia 750004, China; ^3^Center of Laboratory Medicine of the General Hospital, Ningxia Medical University, Yinchuan, Ningxia 750004, China; ^4^Department of Clinical Oncology, Queen Elizabeth Hospital, Kowloon, Hong Kong; ^5^Key Laboratory of Ministry of Education for Conservation and Utilization of Special Biological Resources in Western China, Ningxia University, Yinchuan, Ningxia 750021, China

## Abstract

Lung cancer is a leading cause of cancer mortality worldwide. Several molecular pathways underlying mechanisms of this disease have been partly elucidated, among which the epidermal growth factor receptor (EGFR) pathway is one of the well-known signaling cascades that plays a critical role in tumorigenesis. Dysregulation of the EGFR signaling is frequently found in lung cancer. The strategies to effectively inhibit EGFR signaling pathway have been mounted for developing anticancer therapeutic agents. However, most anti-EGFR-targeted agents fail to repress cancer progression because of developing drug-resistance. Therefore, studies of the mechanisms underpinning the resistance toward anti-EGFR agents may provide important findings for lung cancer treatment using anti-EGFR therapies. Recently, increasing numbers of miRNAs are correlated with the drug resistance of lung cancer cells to anti-EGFR agents, indicating that miRNAs may serve as novel targets and/or promising predictive biomarkers for anti-EGFR therapy. In this paper, we summarize the emerging role of miRNAs as regulators to modulate the EGFR signaling and the resistance of lung cancer cells to anti-EGFR therapy. We also highlight the evidence supporting the use of miRNAs as biomarkers for response to anti-EGFR agents and as novel therapeutic targets to circumvent the resistance of lung cancer cells to EGFR inhibitors.

## 1. Introduction

Lung cancer is a heterogeneous disease, which is the leading cause of cancer-related mortality worldwide. It can be broadly classified into non-small-cell lung cancer (NSCLC) and small cell lung cancer based on clinical, histological, molecular, and endocrinological characteristics. Using histological features, NSCLC can be further subdivided into large-cell carcinoma, bronchoalveolar lung cancer, adenocarcinoma, squamous carcinoma, and mixed histological types (e.g., adenosquamous carcinoma) [1, 2]. NSCLC accounts for more than 85% of all patients with lung cancer. To date, platinum-based doublets remain the mainstay in the treatment of patients with advanced NSCLC [2]. With an increased understanding of the mechanisms underpinning lung cancer development and progression, a number of novel agents specifically targeting oncogenic pathways have been developed and applied to treat lung cancer [3], among which the inhibitor targeting epidermal growth factor receptor (EGFR) signaling cascades is one of the most broadly used agents implemented in clinical practice [3–5].

EGFR is a receptor of tyrosine kinase (RTK), which consists of an N-terminus extracellular ligand-binding site, a hydrophobic transmembrane domain, and a C-terminus intracellular region with tyrosine kinase activity [5]. The EGFR signaling network plays a central role in the growth and maintenance of epithelial tissues. The dysregulation and/or hyperactivation of the EGFR signaling pathway are frequently found in epithelial lung tumor entities, in which the hyperactivated EGFR signaling is associated with advanced lung cancer and poor prognosis [6]. Therefore, EGFR and its downstream signaling components can be used as major targets in developing novel agents for cancer treatment, such as chimeric monoclonal antibodies (cetuximab and panitumumab) [2] and tyrosine kinase inhibitors (TKIs) (gefitinib, erlotinib, and afatinib) [7–9]. However, the clinical benefits of these anti-EGFR agents are often limited, mainly due to the heterogeneity of lung cancer and the drug resistance to anti-EGFR therapy [10]. Consequentially, a large number of studies focus on the mechanisms underpinning the resistance toward anti-EGFR agents.

MicroRNAs (miRNAs) are a class of small noncoding RNAs that act as key posttranscriptional regulators of gene expression. They can functionally impact cell fate determination by regulating the expression of critical proteins; thus miRNAs play a pivotal role in the diverse processes of human cancer, acting as either tumor suppressors or oncogenes [11–13]. Recently, several studies have demonstrated that EGFR is a target of a number of miRNAs, and* vice versa* a mutation or activity of the EGFR signaling pathway can alter the expressions of miRNAs in lung cancer (Table 1) [14–17]. The involvement of miRNAs in the EGFR signaling pathway of lung cancer development and target therapy has recently gained increasing attentions [16]. In this review, we summarize the emerging role of miRNAs as modulators to regulate the EGFR signaling pathway and mainly focus on miRNAs as predictive biomarkers for anti-EGFR therapy and as novel targets to reverse the resistance of lung cancer cells to EGFR inhibitors.

## 2. miRNAs Target EGFR in Human Lung Cancers

**Figure 1:**
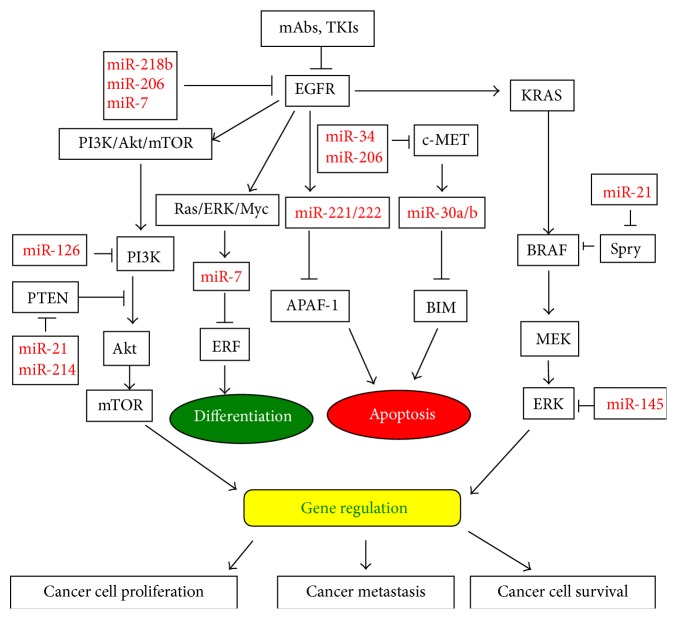
An illustration representing microRNAs (miRNAs) and their targets involved in EGFR signaling pathway in lung cancer and anti-EGFR therapy. The depicted miRNAs target important signaling pathways in lung cancer development and resistance to anti-EGFR agents.

miRNAs have gained increasing interest owing to their widespread occurrence and diverse functions as regulatory molecules in many signaling pathways, including the EGFR signaling pathway. Accumulating evidence has revealed that miRNAs are oncomirs or tumor suppressors by targeting the EGFR signaling pathway in different types of cancer. Table 1 lists several miRNAs that regulate the EGFR signaling pathway in lung cancer and Figure 1 shows the miRNAs that target the EGFR signaling pathway.

Using bioinformatics tools, Chan et al. predicted 138 miRNAs that potentially target EGFR in NSCLC, some of these miRNAs were confirmed experimentally [18]. Among these miRNAs, miR-7 has been demonstrated to downregulate EGFR in different cancer cells. Mechanistically, miR-7 could directly target EGFR by binding three seeding sequences in human EGFR mRNA 3′-UTR [19, 20]. Apart from its ability to directly target EGFR mRNA, miR-7 can also target several downstream effectors of the EGFR signaling pathway, including the proto-oncogene serine/threonine protein kinase RAF-1, protein kinase B Akt, and extracellular signal-regulated kinase 1/2 (ERK1/2), sequentially inhibits cancer cell migration, invasion, and metastasis [16, 20]. In lung cancer, miR-7 was upregulated in 60% of NSCLC fine-needle aspirates, which could be induced by both wild type and mutant EGFR L858R, and plays an oncogenic role by activating the rat sarcoma viral oncogene homolog (RAS)/ERK/c-Myc signaling axis to promote lung carcinogenesis by repressing the transcriptional regulator Ets2 transcriptional repression factor (ERF) [16, 21]. In this context, an activation of EGFR or ectopic expression of RAS and c-Myc could induce miR-7 transcription in an extracellular signal-regulated kinase- (ERK-) dependent manner. This notion was supported by findings of that c-Myc bound to the miR-7 promoter and enhanced its activity, and an enforced expression of miR-7 promoted cell growth and tumor formation in lung cancer cells and significantly increased the mortality of nude mice orthotopically implanted with lung cancers. Molecular analysis further revealed that miR-7 could directly target ERF, a seeding sequence of miR-7 that was confirmed in the coding sequence of ERF, suggesting that miR-7 may act as an important regulator of EGFR-mediated oncogenesis and can be served as a novel prognostic biomarker and therapeutic target in lung cancer [21].

miRNA profiling of lung cancer cell lines and lung tissues has demonstrated that miRNAs are emerging as unique effectors of the EGFR signaling pathway in lung cancer, in which miRNAs are correlated with the expression of EGFR and/or the EGFR mutant status or signaling activities [22, 23]. Analyzing miRNA expression profiling of lung cancer, Dacic et al. observed a correlation of miRNAs with mutational status of EGFR in lung adenocarcinomas, in which miR-155 was upregulated only in EGFR/KRAS-negative samples, and miR-25 was upregulated only in EGFR-positive group and miR-495 was upregulated only in KRAS-positive adenocarcinomas. Conversely, let-7g was dramatically downregulated in EGFR/KRAS negative adenocarcinomas [23]. Such a correlation was also found in other mRNAs. For examples, miR-542-5p could downregulate EGFR mRNA and protein expression in human lung cancer H3255, A549, and HCC827 cells and inhibit the growth of these cancer cells. Interestingly, an inverse correlation of miR-542-5p transcript and EGFR protein levels was found in human lung cancer tissues [24]. Such an inverse correlation of miRNA expression and the EGFR signaling pathway was also found in miR-133a whose expression was negatively correlated with cell invasiveness in lung cancer cell lines, by targeting insulin-like growth factor 1 receptor (IGF-1R), TGF-beta receptor type-1 (TGF*β*R1), and EGFR [25]. Similarly, Chan et al. also demonstrated that miR-146a inhibited cell growth and induced cell apoptosis by suppressing the EGFR downstream signaling components and the migratory capacity in various NSCLC cell lines (H358, H1650, H1975, HCC827, and H292), through an EGFR mutation status independent mechanism of directly targeting the EGFR and nuclear factor kappa beta (NF-*κ*B) signaling pathways [18].

In line with the regulatory role of miRNAs in the EGFR signaling pathway, the aberrant expression and/or mutation(s) of EGFR may also alter the expression of miRNAs in lung cancer. For instance, the expression of some miRNAs, such as miR-21 was altered more remarkably in a lung cancer with EGFR mutations relative to those without these mutations [26], suggesting that the EGFR signaling pathway is not only regulated by tumor-suppressive miRNAs, but also has potential to regulate some miRNAs acting as oncogene. In a recent study by Guo et al., the authors found that aberrant activation of the EGFR signaling pathway downregulated miR-145 expression in NSCLC, an addition of EGFR inhibitor AG1478 that could restore the expression of miR-145 in lung cancer cells [27]. Using an Agilent microarray, Bjaanaes et al. examined the expression of miRNAs in 154 surgically resected lung adenocarcinomas and 20 corresponding normal lung tissue samples; they found that 129 miRNAs were strikingly differentially expressed in lung adenocarcinomas in comparison with normal lung tissues, among which 17 miRNAs were differentially expressed between tumors with EGFR-mutation and wild-type [22]. These studies imply a feedback regulatory mechanism between the EGFR signaling pathway and miRNAs in the development and progression of lung cancers.

## 3. miRNAs Alter EGFR-TKI Responses in Lung Cancer

Targeting therapy to the EGFR signaling pathway leads to development of EGFR tyrosine kinase inhibitors (TKIs), namely, gefitinib, erlotinib, and afatinib, for the treatment of patients with NSCLC who have EGFR mutations. Different from wild-type EGFR, mutations of EGFR may confer hypersensitivity to TKIs in advanced NSCLC [28], since cells with mutant EGFR transduce survival signals but have no effect in proliferative signals [8]. However, the clinical benefit of TKIs was limited as patients eventually develop resistance to these agents. 70% of this acquired resistance (AR) may be caused by a secondary mutation in the EGFR gene, such as T790M or amplification of the proto-oncogene hepatocyte growth factor receptor (c-MET). In gefitinib or erlotinib resistant tumor samples, about 50% samples have been found to bear T790M mutation and the other 20% cases have c-MET amplification. Mechanically, T790M can increase GTP affinity in the tyrosine kinase domain or block TKI binding to the tyrosine kinase domain of EGFR [29]. Other mechanisms, including the involvement of Anexelekto- (Axl-) kinase and a number of miRNAs in the AR of lung cancer to TKIs [10, 16], and several miRNAs have been demonstrated to be associated with EGFR mutations in lung cancer (Table 2) [30].

Increasing number of studies has revealed a correlation of the clinical responses to TKIs and the expressions of miRNAs. For example, loss of heterozygosity (LOH) at miR-128b is one of the most frequent genetic events in lung cancer. Weiss et al. found that LOH at miR-128b was frequent detected in lung cancer tissues and was positively correlated with clinical response and survival to gefitinib treatment [15]. Other studies showed that the restoration of miR-145 and miR-7 inhibited cancer cell growth in lung adenocarcinoma patients with EGFR activating mutation and could effectively target EGFR addicted and EGFR-TKI resistant tumors [21, 31]. In a recent study, Garofalo et al. demonstrated that RTK of EGFR and c-MET could induce miR-30b/30c/221/222 expressions, and an upregulation of miR-30b/30c/221/222 induced resistance to gefitinib in lung cancer cells by the regulation of BCL2-like 11 (BIM), phosphatase and tensin homolog (PTEN), and apoptotic peptidase activating factor 1 (APAF-1) expressions. In contrast, miR-103/203 could induce gefitinib resistant cell apoptosis and promote mesenchymal to epithelial transformation (MET) by targeting protein kinase C varepsilon (PKC-*ε*) and sarcoma viral oncogene homolog (SRC), but the ectopic expressions of miR-30b/30c/221/222 conferred resistance to TKIs [32]. Such AR to TKIs could also be induced by miR-214 and miR-21 in lung cancer cells [33]. miR-214 expression was elevated in gefitinib resistant HCC827 lung cancer cells (HCC827/GR), which was inverse with PTEN expression. A knockdown of miR-214 in HCC827/GR showed a restoration of PTEN expression and resensitized HCC827/GR to gefitinib. Similar to miR-21, which was also more aberrantly expressed in EGFR-TKI-resistant lung cancer cell line PC9R relative to its parent cell PC9. The increased level of miR-21 was inversely correlated with the abundance of PTEN and programmed cell death protein 4 (PDCD4) proteins and positively correlated with the phosphatidylinositol-3-kinase (PI3K)/Akt signaling pathway. An inhibition of miR-21 induced apoptosis in PC9R cells and suppressed tumor growth in nude mice treated with EGFR-TKI. Clinically, circulating miR-21 level in EGFR-TKI-treated NSCLC patients was significantly higher at the time of acquiring resistance over the baseline [26, 33]. Recent studies in lung adenocarcinoma of female nonsmokers also revealed that the expression of miR-183-3p, miR-195, and miR-122 was in plasma and associated with EGFR mutations in lung cancer [34, 35].

Intriguingly, several tumor-suppressive miRNAs exhibited an ability to enhance the cytotoxicity of EGFR-TKI to lung cancer cells, such as miR-126, miR-145, and miR-146a [40, 54, 61]. Lung cancer cells H460 and A549 are significantly resistant to gefitinib, forced expression of miR-126 and miR-145 showed an inhibited growth of cells and tumor xenografts and an enhanced cytotoxicity induced by gefitinib [54]. miR-146a is an extensively studied tumor-suppressive miRNA in many types of cancer, the polymorphism of the rs2910164CNG in pre-miR-146a was recently identified to be associated with the genetic susceptibility to lung cancer development in a Korean population [61]. In addition, a downregulation of miR-146a was reported in lung cancers, overexpression of miR-146a was found to suppress cell growth and migration, induce cellular apoptosis, and inhibit the EGFR downstream signaling components in lung cancer cell lines H358, H1650, H1975, HCC827, and H292. Importantly, forced expression of miR-146a could enhance the ability of EGFR-TKIs (gefitinib, erlotinib, and afatinib) and monoclonal antibody (cetuximab) to inhibit cell proliferation by targeting of the EGFR and NF-*κ*B signaling pathways [40].

The activation of c-MET is associated with both primary and acquired resistance to EGFR-TKIs in patients with NSCLCs [62]. Both EGFR and c-MET are RTKs that have been implicated in tumor progression as regulators of miRNA cluster 23a/27a~24-2 in lung cancer, in which miR-27a can regulate both c-MET and EGFR [36]. Such a dual inhibitory role of miRNAs to the c-MET and EGFR oncogenic signaling pathways was also recently identified for miR-206 in lung squamous cell carcinoma [45]. Therefore, simultaneous inhibition of these RTKs might improve disease treatment. The evidence of miRNA participating in the EGFR/c-MET network in lung cancer thus provides a new clue to overcoming EGFR-TKI resistance in lung cancer [63].

## 4. miRNAs as Biomarkers for Predicting EGFR-TKI Response in Lung Cancer

Given the fact that only small portion of patients with lung cancer benefit from a treatment of EGFR-TKIs, the benefits of these agents to patients are ultimately limited by the emergence of drug resistance [64]. Therefore, great efforts have been made to identify new biomarkers for predicting responses to TKI treatment in lung cancer. The ability to alter EGFR-TKIs responses makes miRNAs as potential predictive biomarkers for EGFR-TKIs in lung cancer treatment, and several miRNAs could be served as biomarkers to predict response to EGFR-TKIs in lung cancer patients have been recently well documented (Table 3) [9, 65].

In a miRNA profiling analysis with retrospective cohorts consisted of 128 radically resected NSCLC patients (60 were EGFR mutation positive, 68 were negative, and 32 healthy controls), Shen et al. found that the expression of miR-21 and miR-10b in radically resected NSCLC patients with EGFR mutation were much higher relative to those without mutation. A Cox proportional-hazards regression analysis further demonstrated that a reduced expression of miR-21 was associated with a significant improvement in overall survival in patients treated with gefitinib; that is, a patient who had upregulated miR-21 expression might have poor overall survival, but a better response to gefitinib. This data suggests that miR-21 expression may be an independent predictor of the response to gefitinib in lung cancer [30]. Of interest, an aberrant expression of miR-21 was also significantly correlated with platinum-based chemotherapy resistance in NSCLC patients, and an increased miR-21 expression was associated with the shorter disease-free survival [62].

Other miRNAs that targeting the EGFR signaling pathway, such as miR-128b [15], miR-30b, and miR-30c [32], were also significantly correlated with clinical EGFR-TKI responses in lung cancer, in which LOH at miR-128b is frequent in NSCLC [15], and the expressions of miR-30b and miR-30c have been reported to be prognostic predictors in NSCLC patients who underwent first line treatment with EGFR-TKIs [50]. Gu et al. retrospectively examined expression of miR-30b and miR-30c in 41 NSCLC samples of patients who used TKIs as first line of therapy. They found that there is a significant correlation of miR-30b and miR-30c levels and the short-term TKI responses, suggesting that miR-30b and miR-30c may be useful in predicting TKI response in NSCLC patients [50]. Another study using lung epithelial cancer cell line model has identified 13 miRNA genes to predict response to EGFR inhibitors, among which the miR-200c was able to target epithelial-to-mesenchymal transition (EMT) transcription factor, zinc finger homeodomain enhancer-binding protein 1 (ZEB1) and altered the sensitivity to erlotinib, and migration in lung cells [56]. The transforming growth factor-*β* (TGF-*β*) is able to induce EMT in cancers [49, 57]. A negative regulator of EGFR, mitogen-inducible gene 6 (MIG6) is a target of miR-200, the ratio of the expression of MIG6/miR200c was found to be tightly correlated with EMT and resistance to erlotinib in lung cancer* in vivo*, in which the MIG6 (mRNA)/miR200 ratio was inversely correlated with response to erlotinib, indicating that the ratio of MIG6/miR200 may be a predictive biomarker of the response of lung cancer to EGFR-TKIs [57].

## 5. miRNAs as Therapeutic Targets for Sensitizing EGFR-TKI-Resistant Lung Cancer

The emerging role of miRNAs in regulation the EGFR signaling pathway and therapeutic responses to EGFR-TKIs has provided a new avenue for developing novel agents and approaches to resensitize TKI resistance and improve the overall clinical outcomes of TKI-treatment in patients with lung cancer. Clinically, majority of EGFR-TKI resistance is induced by a secondary T790M mutation of EGFR or c-MET. In this regard, a T790M mutation is able to enhance the GTP affinity and block TKI binding to the tyrosine kinase domain of EGFR [29]. Therefore, a strategy by targeting G protein-coupled receptor or c-MET may reverse lung cancer cells to EGFR-TKI resistance. In order to overcome the EGFR-TKI resistance in T790M mutant NSCLC treatment, Rai et al. delivered miR-7 expressing plasmid to NSCLC cells and xenografts by liposomal transfection and found that the miR-7 could inhibit the growth of both TKI sensitive and resistant NSCLC cells* in vitro* and* in vivo* [59]. This finding was supported by a late study using Lewis lung cancer (3LL) cells with a downregulated miR-7; this study demonstrated that a restoration of miR-7 inhibited 3LL cell proliferation, induced cell apoptosis* in vitro,* and reduced tumorigenicity* in vivo* by targeting the EGFR signaling pathway [60].

Gao et al. recently identified miR-138-5p was strikingly downregulated, which was inversely correlated with the expression of G protein-coupled receptor 124 (GPR124) in a gefitinib-resistant lung cancer cell line PC9GR. Bioinformatics analysis suggested that the GPR124 was a direct target of miR-138-5p, which was further validated experimentally. Intriguingly, forced expression of miR-138-5p was sufficient to resensitize the PC9GR cells and gefitinib resistant NSCLC H1975 cells to gefitinib, and knockdown of GPR124 with small RNA mimics exhibited similar effects of miR-138-5p, suggesting that the acquired gefitinib resistance was in part attributed by a downregulation of miR-138-5p and that restoration of miR-138-5p level might be a potential therapeutic approach for sensitizing gefitinib resistance in NSCLC [53]. Using a similar approach, Zhou et al. found that an ectopic expression of miR-34a could inhibit cell growth and induce apoptosis in hepatocyte growth factor- (HGF-) induced gefitinib-resistant HCC827GR and PC-9GR lung cancer cells and in HGF-induced gefitinib resistant mouse xenograft model, partly by targeting MET [51]. In this context, an upregulation of HGF has been demonstrated as an important mechanism involved in the AR to EGFR-TKIs by activation of PI3K/Akt pathway through phosphorylation of c-MET [32, 36, 62]. This notion was supported by the evidence of that the total and phosphorylated of c-MET proteins were partially decreased in gefitinib-sensitive HCC827 and PC-9 cells, but the total and phosphorylated status of the downstream PI3K/Akt or ERK signaling pathway was not affected by the transfection of miR-34a. This result indicated that miR-34a had an inhibitory effect on MET rather than its downstream signaling components, which also implies that the tumor suppressive effects of miR-34a alone in gefitinib-sensitive EGFR mutant NSCLC cells might mainly be dependent on mechanisms other than c-MET inhibition. This was different from in the gefitinib-resistant HCC827GR and PC-9GR cells, in which a combination of miR-34a and gefitinib could efficiently induced cell death and apoptosis with an inhibition of the phosphorylation of c-MET, EGFR, Akt, and ERK [51]. Such a synergistic effect between the miR-34a and EGFR-TKIs was also reported in study of a combination of erlotinib and miR-34a in NSCLC cells with primary and acquired erlotinib resistance, in which a strong synergistic interaction between the erlotinib and miR-34a mimics was observed [51]. These studies clearly suggest that a synergistic strategy using miRNAs and TKIs in a combination may effectively reverse the EGFR-TKI resistance in lung cancer treatment.

Both experimental and preclinical studies have demonstrated that persistent activation of PI3K/Akt and/or Ras/Erk pathways is associated with EGFR-TKI resistance in NSCLC, in which they play pivotal roles in TKI sensitivity [66]. Indeed, the overexpression of miR-21 led to a significant decrease of gefitinib sensitivity in PC9 lung cancer cells through a mechanism by inhibiting PTEN expression and activating the Akt/Erk signaling pathway, while knockdown of miR-21 dramatically reversed gefitinib sensitivity in PC9GR cells by upregulating PTEN and inactivating the Akt/Erk pathway, suggesting modulation of miR-21/PTEN expression may be a promising strategy for resensitizing EGFR-TKI resistance in NSCLC [33].

Apart from targeting the PI3K/Akt/Ras/Erk pathway, EMT is involved in the AR to therapy, which is often activated during the progression of lung cancer [52, 56, 57, 67]. Several lines of evidence have demonstrated that miRNAs are involved in MET and reverse EGFR-TKI resistance in NSCLC [55, 57, 67]. For instances, miR-147 was downregulated in NSCLC, and overexpression of miR-147 could induce the MET of lung cancer cells, sequentially resensitize the resistance to EGFR-TKIs by inhibiting the Akt signaling pathway, and the MET phenotype of lung cancer cells could be attenuated by TGF-*β* [55]. Exposure NSCLC cells with EMT to TGF-*β*1 was found to significantly induce miR-134/487b/655 cluster by targeting membrane-associated guanylate kinase, WW domain- and PDZ domain-containing protein 2 (MAGI2), a scaffold protein required for PTEN. Ectopic expression of miR-134 and miR-487b enhanced the EMT potential and the drug resistance to gefitinib of the cells, whereas reduction of the transcripts of these miRNAs led to an inhibition of EMT process and a restoration of drug sensitivity of TGF-*β*1-induced resistance to gefitinib, implying that the miR-134/miR-487b/miR-655 cluster may be a novel therapeutic target in patients with advanced lung adenocarcinoma [49]. By modulating EMT-regulating miRNAs, Ahmad et al. demonstrated that both specific siRNA to the Hedgehog (HH) signaling pathway and GDC-0449 (a small molecule antagonist of G protein coupled receptor smoothened in the HH pathway) were able to resensitize TGF-*β*1-induced erlotinib resistant A549 (A549M) cells with an upregulation of miR-200b and let-7c. Ectopic expression of these miRNAs also led to diminish the erlotinib resistance of A549M cells [52]. In another study, Cufí et al. discovered that flavonolignan silibinin could suppress the EMT-driven erlotinib resistance by restoring a high miR-21/low miR-200c signature in EGFR-mutant NSCLC xenografts [67]. A combination of erlotinib and silibinin led a completely abrogate tumor growth in the NSCLC xenograft model. Mechanistically, the silibinin could fully restore the EMT-related high miR-21/low miR-200c signature and inhibit the expression of mesenchymal markers snail family zinc finger 1 (SNAI1), ZEB, and N-cadherin in erlotinib-refractory tumors. In addition, the silibinin was sufficient to fully activate a reciprocal c-MET in erlotinib-refractory cells [67].

In addition to T790M and c-MET amplification, Axl kinase is found to be upregulated in humans with acquired resistance to EGFR-TKI, and the involvement of Axl kinase in acquired resistance of NSCLC to RGFR-TKIs gefitinib or erlotinib has been reported [38, 58, 68]. In order to interrogate the role of miRNAs in the Axl-mediated acquired TKIs resistance in lung cancer, Wang et al. identified a panel of Axl kinase-altered miRNAs in lung cancer cells and experimentally validated that the Axl-induced miR-374a and miR-548b play a crucial role in cell cycle arrest, gefitinib-induced apoptosis, and EMT of gefitinib-resistant lung cancer cells by targeting Wnt5a and CCNB1 genes, respectively. Clinically, a high expression of Axl and miR-374a and low abundance of miR-548b are associated with poor disease-free survival. These observations suggest a promising strategy by targeting miRNAs to reverse gefitinib resistance in NSCLC with high expression of Axl [58, 68].

## 6. Perspectives and Challenges

As a class of regulators at the posttranscription level, miRNAs display different expression patterns in various types of cancer, in which some miRNAs are dysregulated and they play crucial roles in the initiation, progression, and therapeutic responses of cancer. The involvement of miRNAs in the mutant status of EGFR and the emerging role of these molecules in the regulation of the EGFR signaling pathway and drug resistance to anti-EGFR agents have made miRNAs potential biomarkers for the diagnosis and prognosis of lung cancer, as well as potential predictive markers for the therapeutic outcome using anti-EGFR agents or regimens. In a therapeutic standpoint, in addition to restore the functions of tumor suppressor genes or inhibiting oncogenes by targeting miRNAs in anticancer therapy, the modulation of miRNA profiles is also a plausible therapeutic strategies to resensitizing chemoresistance in cancer treatment. In terms of anti-EGFR therapy, an intervention of specific miRNAs that involved in the EGFR signaling pathway and/or EGFR-TKI resistance has shown a promising effect to reverse the resistance of lung cancer cells to anti-EGFR therapy by enhancing the sensitivity of tumor cells to chemotherapy or inhibiting cancer cell stemness.

Although recent studies in lung cancer and miRNAs have significantly extended our understanding of the EGFR signaling pathway and its involvement in the pathogenic processes of lung cancer, our understanding of the underlying mechanisms that integrate the activity of this pathway remains fragmentary. Therefore, intensively exploring the regulatory roles of miRNAs in this pathway may contribute to the possible implementation of miRNAs as predictive and prognostic biomarkers [69, 70]. Particularly, the application of miRNAs as predictive biomarkers may also be beneficial for predicting therapeutic response to anti-EGFR agents in advanced lung cancer patients and lead to a higher level of personalized therapy. However, challenges for the development of miRNA in therapy remain to be addressed; these include tissue specific delivery, potential off-target effects, and safety. An improvement of the specificity of miRNAs and the development of efficient systemic delivery approaches will facilitate the use of miRNAs for the treatment of patients with lung cancer.

## Figures and Tables

**Table 1:** MicroRNAs that target EGFR signaling pathway involved in lung cancer.

MicroRNA(s)	Regulation	Potential function(s)	Reference(s)
let-7g	Down	Dramatically downregulated in EGFR/KRAS negative lung adenocarcinomas	[23]
miR-7	Down	Inhibits EGFR-PI3K-AKT signaling and reverses radio-resistance in various cancer cells	[20]
miR-27a	Down	Directly targets EGFR and contributes to mutant p53 gain-of-function	[36, 37]
miR-34a	Down	Regulates Axl receptor tyrosine kinase by targeting SIRT1 and MEK1	[38]
miR-128b	Down	Directly regulates EGFR expression in NSCLC	[15, 18]
miR-133a	Down	Repress EGFR signaling by directly targeting IGF-1R, TGF*β*R1, and EGFR	[25]
miR-133b	Down	Suppresses EGFR pathway signaling and enhances susceptibility to EGFR-TKI in lung cancer cells by directly targeting EGFR	[39]
miR-145	Down	Negatively regulates EGFR expression in lung cancer cells	[31]
miR-146a	Down	Inhibit EGFR in NSCLC cancer cells	[40, 41]
miR-146b-5p	Down	Suppressed EGFR expression in glioblastoma cell lines	[42]
miR-200	Down	Regulates EMT in anaplastic thyroid cancer cells and bladder cancer cells and reverses resistance of EGFR therapy	[43, 44]
miR-206	Down	Suppresses EGFR signaling in squamous lung cancer cells by directly targeting EGFR and MET	[45]
miR-542-5p, 1203, 1237, 541, 1911	Down	Downregulates EGFR in human lung cancer cells	[20, 24]
miR-21	Up	Regulate the EGFR/AKT pathway in a PTEN independent manner	[46]
miR-24	Up	Activates EGFR signaling by targeting PTPN9 and PTPRF	[47]
miR-25	Up	Upregulated in EGFR positive lung cancer	[23]
miR-214	Up	Regulate acquired resistance to EGFR-TKIs in cancer cells through a PTEN/AKT signaling pathway	[48]

**Table 2:** Alteration of EGFR mutation related microRNAs in lung cancer.

MicroRNA(s)	Regulation	Chromosome locus	Reference(s)
miR-10b	Up	2q31.1	[30]
miR-21	Up	17q23.1	[26, 30]
miR-122	Up	18q21.3	[34]
miR-134/487b/655 cluster	Up	14q32	[49]
miR-183-3p	Up	7p32	[35]
miR-200b	Up	1p36.33	[30]
miR-210	Up	11p15.5	[30]
miR-30a	Down	6q13	[30]
miR-30b	Down	8q24.22	[30, 50]
miR-30c	Down	1p34.2	[50]
miR-34a	Down	1p36.23	[51]
miR-126	Down	9q34.3	[30]
miR-145	Down	5q32-33	[31]
miR-451	Down	17q11.2	[30]

**Table 3:** MicroRNAs regulate chemoresistance in lung cancer.

MicroRNA(s)	Regulation	Agent	Target(s)	Reference(s)
let-7	Down	Erlotinib	Hedgehog	[52]
miR-34	Down	Gefitinib	c-MET/HGF	[51]
miR-103	Down	Gefitinib	PKC-*ε*	[32]
miR-128b	Down	Gefitinib	EGFR	[15]
miR-138-5p	Down	Gefitinib	GPR124	[53]
miR-145	Down	TKIs	ERK, AKT, OCT4, c-MYC, EGFR, and NUDT1	[31, 54]
miR-146a	Down	TKIs	EGFR and NF-*κ*B	[40]
miR-147	Down	Gefitinib	ZEB1 and AKT	[55]
miR-200	Down	Erlotinib	Hedgehog, MIG6, and TGF*β*1	[52, 56, 57]
miR-203	Down	Gefitinib	SRC	[32]
miR-424	Down	TKIs	Not applicable	[15]
miR-548b	Down	TKIs	CCNB1	[58]
miR-7	Up	TKIs	EGFR, RAF1, and IRS-1	[21, 59, 60]
miR-21	Up	Gefitinib	PTEN, MDR1, Bcl-2, and PDCD4	[33]
miR-30b/30c	Up	Gefitinib	BIM	[32]
miR-126	Up	Gefitinib	AKT, EGFL7, PI3KR2, ERK, CRK, and VEGF	[54]
miR-134/487b/655 cluster	Up	Gefitinib	MAGI2	[49]
miR-221/222	Up	Gefitinib	APAF-1	[32]
miR-214	Up	TKIs	PTEN, MAPK, and p38	[46]
miR-374a	Up	TKIs	Wnt5a	[58]

## Abbreviations

AKT: Protein kinase B
APAF-1: Apoptotic peptidase activating factor 1
Axl: Anexelekto
BIM: BCL2-like 11
CCNB1: Cyclin B1
CCND1: Cyclin D1
c-MET: The transmembrane tyrosine kinase cell surface receptor for HGF
c-Myc: A regulator gene that codes for a transcription factor
CRK: CT10 regulator of kinase (Crk)
EGFR: Epidermal growth factor receptor
EMT: Epithelial to mesenchymal transition
ERF: Ets2 transcriptional repression factor
ERK: Extracellular signal-regulated kinase
GPR124: G protein-coupled receptor 124
HGF: Hepatocyte growth factor
HH: Hedgehog
IGF1R: Insulin-like growth factor 1 receptor
IRS-1: Insulin receptor substrate-1
K-Ras: Kirsten rat sarcoma viral oncogene homolog
LOH: Loss of heterozygosity
MAGI2: Membrane-associated guanylate kinase, WW, and PDZ domain-containing protein 2
MAPK: Mitogen-activated protein kinase
MDR-1: Multidrug resistance 1
MET: Mesenchymal to epithelial transition
MIG6: Mitogen-inducible gene 6
NF-*κ*B: Nuclear factor kappa beta
NSCLC: Non-small-cell lung cancer
NUDT1: Nucleoside diphosphate linked moiety X-type motif 1
PDCD4: Programmed cell death protein 4
PDZ domain: An interaction motif that recognizes and binds the C-terminal peptides of target proteins
PI3K: Phosphatidylinositol-3-kinase
PKC-*ε*: Protein kinase C varepsilon
PTEN: Phosphatase and tensin homolog
PTPN9: Phosphatases tyrosine-protein phosphatase nonreceptor type 9
PTPRF: Receptor-type tyrosine-protein phosphatase F
RAF-1: Proto-oncogene serine/threonine protein kinase
RAS: Rat sarcoma viral oncogene homolog
RTK: Receptor of tyrosine kinase
SIRT1: Sirtuin 1
SNAI1: Snail family zinc finger 1
SRC: Sarcoma viral oncogene homolog
TGF*β*1: Transforming growth factor beta 1
TGF*β*R1: Transforming growth factor beta receptor 1
VEGF: Vascular endothelial to growth factor
Wnt: Wingless-type MMTV integration site family
WW domain: A short conserved protein domain mediates interactions with protein ligands
ZEB: Zinc finger homeodomain enhancer-binding protein.
